# Authorized Traffic Controller Hand Gesture Recognition for Situation-Aware Autonomous Driving

**DOI:** 10.3390/s21237914

**Published:** 2021-11-27

**Authors:** Ashutosh Mishra, Jinhyuk Kim, Jaekwang Cha, Dohyun Kim, Shiho Kim

**Affiliations:** Yonsei Institute of Convergence Technology, Yonsei University, Incheon 21983, Korea; ashutoshmishra@yonsei.ac.kr (A.M.); jinhyuk.kim@yonsei.ac.kr (J.K.); chajae42@yonsei.ac.kr (J.C.); kimdh5032@yonsei.ac.kr (D.K.)

**Keywords:** 3D hand-pose modeling, authorized traffic controller, autonomous vehicle, irregular situation, situation-aware, traffic control hand gesture recognition

## Abstract

An authorized traffic controller (ATC) has the highest priority for direct road traffic. In some irregular situations, the ATC supersedes other traffic control. Human drivers indigenously understand such situations and tend to follow the ATC; however, an autonomous vehicle (AV) can become confused in such circumstances. Therefore, autonomous driving (AD) crucially requires a human-level understanding of situation-aware traffic gesture recognition. In AVs, vision-based recognition is particularly desirable because of its suitability; however, such recognition systems have various bottlenecks, such as failing to recognize other humans on the road, identifying a variety of ATCs, and gloves in the hands of ATCs. We propose a situation-aware traffic control hand-gesture recognition system, which includes ATC detection and gesture recognition. Three-dimensional (3D) hand model-based gesture recognition is used to mitigate the problem associated with gloves. Our database contains separate training and test videos of approximately 60 min length, captured at a frame rate of 24 frames per second. It has 35,291 different frames that belong to traffic control hand gestures. Our approach correctly recognized traffic control hand gestures; therefore, the proposed system can be considered as an extension of the operational domain of the AV.

## 1. Introduction

**Figure 1:**
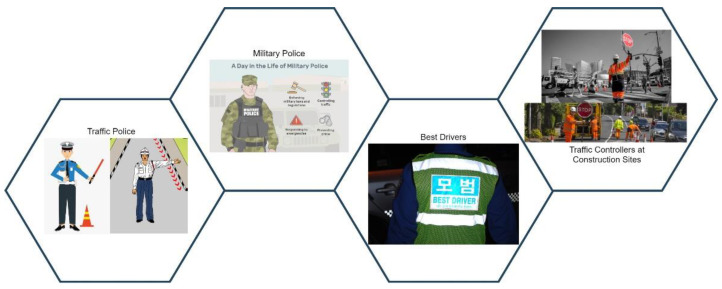
Examples of the possible controllers directing on-road traffic. Best drivers are considered as authorized traffic controllers (ATC) in South Korea.

Traffic police make traffic control hand gestures to control the flow of vehicles and traffic on the road for human safety. However, there are other people or objects (e.g., traffic mannequins, traffic robots, etc.) that use hand gestures to participate in the task of traffic directing. Such possible authorized controllers include the traffic police, private traffic controllers, best drivers (only in Korea), construction workers, and military police (shown in Figure 1). Therefore, we call such controllers authorized traffic controllers (ATCs). ATCs are the top authority in directing on-road traffic. 

A human driver has the inherent ability of situational awareness. Therefore, human drivers tend to follow the directions of ATCs in the case of an irregular situation. Similarly, autonomous driving (AD) should also involve a human-level understanding of situation-aware traffic gesture recognition. In particular, Level 3 and higher autonomous vehicles (AVs) require an understanding of traffic control hand gestures for their seamless transportation [1,2]. Furthermore, there are certain hand gestures on a road that are not traffic-control hand gestures [3]. These gestures are imparted by humans on roads; however, such hand gestures do not have any traffic directional intentions. They create severe confusion in deep-learning-based automated driving systems (ADSs) for Level 3 and higher AVs [4]. Generally, humans (pedestrians, travelers, shopkeepers, etc.) on the road inadvertently use hand gestures very similar to traffic control hand gestures in their daily life. They never intend to impose such gestures (on the road) to direct traffic. However, these situations result in serious confusion in front of intelligent ADSs of Level 3 and higher AVs.

**Figure 2:**
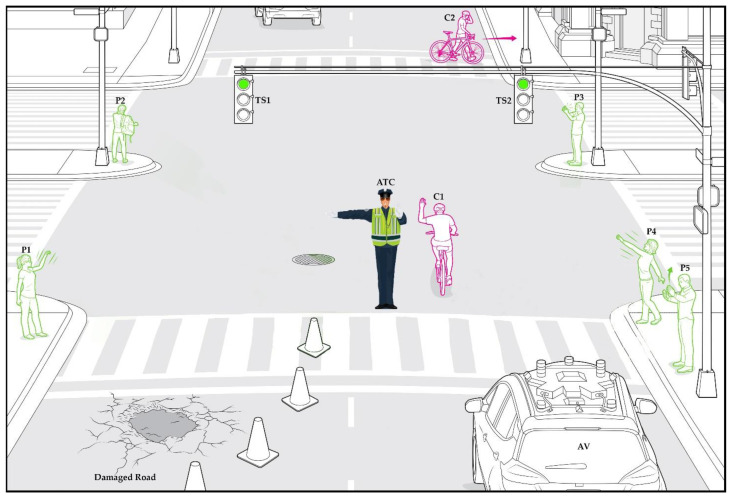
Illustration of an irregular situation with a few commonly occurring scenarios on the road.

Figure 2 represents a few commonly occurring on-road scenarios. In this scene, an ATC directs a vehicle to avoid a large pothole. Here, the traffic signals (TS) are green, indicating that the AV can go through the intersection. However, the ATC is showing a STOP hand gesture. This is an example of an irregular situation, in which the ATC supersedes the TS for directing road traffic. A human driver understands the situation and intends to follow the ATC in such cases; therefore, the AV should also follow the instructions given by the ATC. 

In other scenes, pedestrians (P) and cyclists (C) are imparting hand gestures. In one scenario, a pedestrian (P1) waves his hands to communicate with a friend (P4). Another person (P3) makes sounds using their hands for another pedestrian (P2). At a street corner, P5 is checking a cellphone and accordingly performing hand gestures. A cyclist (C1) is waving his hand. These circumstances create confusion for AVs. In this combined example, only a few gestures are related to traffic gestures. Interestingly, a human driver can easily discriminate in these situations and follow only the correct hand gestures related to traffic control; however, the same situation becomes critical for vision-based intelligent ADS systems in AVs. It becomes a more peculiar and difficult circumstance in the case of AVs higher than Level 3. 

Gloves impose another difficulty in recognizing hand gestures, creating difficulties in accurate hand-gesture recognition. Generally, hand gestures are recognized using a hand landmark model. Gloves cover these hand landmarks and cause difficulties in hand-gesture recognition.

**Figure 3:**
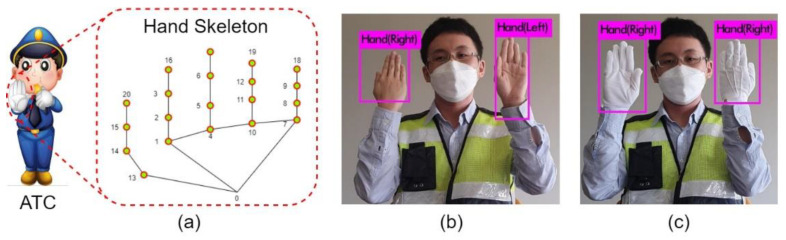
Illustration of the traffic control hand gesture and the associated problem with gloves: (**a**) hand landmark skeleton of the authorized traffic controller (ATC). Hand traffic gesture detection: (**b**) without gloves and (**c**) with gloves.

Figure 3 represents the hand landmark model and the problem associated with hand gestures with gloves. Gloves veil the necessary hand textures and landmarks, which eventually cause confusion, for example, the front or back of the hand. The hand detector correctly identified the hands in Figure 3b; however, it got confused and wrongly predicted the hands in Figure 3c. This is because of the gloves worn on the hands. The model was able to predict hands using the hand skeleton information; however, the gloves covered the features required for prediction. Eventually, the model predicted the same for both hands with gloves. This confusing situation can cause a severe problem as there is a big difference in the meaning of the front and back sides of the hand palm. Section 2 provides the significance of hand palms in traffic-control gestures (with their meaning).

In addition, Level 5 vehicles do not have an active human driver [5,6]. This requires a robust solution for directing the AV safely and seamlessly, under such challenging circumstances. The vehicle should discriminate between potential traffic control hand gestures and other gestures of pedestrians. Therefore, AVs from Level 3 to Level 5 must have the following capability to avoid calamity during irregular situations:

1. ATC recognition;
2. Correct recognition of traffic control hand gestures (even with the palm in gloves).

Therefore, an extension of the operational design domain (ODD) is required for such AVs. In this study, we considered these two problems, and proposed a traffic control gesture recognition system to address these issues. We combined the traffic control hand gestures used in different countries to obtain a uniform traffic control gesture. Our system uses 3D hand pose modeling of authorized traffic controllers. Furthermore, an efficient classifier recognizes traffic-control gestures. Our main contributions are as follows:

∎ Detecting ATCs among other persons on the road;
∎ Detecting the hand and palm of the ATC;
∎ Modeling the hand and palm of the ATC in 3D (to avoid confusion between the back and front of the palm);
∎ Classifying traffic control hand gestures using 3D hand features.

## 2. Materials

Numerous researchers have focused on identifying traffic gestures. This is very important for AVs. AVs below Level 4 use a driver assistance system to assist drivers by providing recommendations. Advanced driver assistance systems (ADASs) are used for such recommendations. However, at Level 3 and higher, it becomes necessary to direct the AV properly on the road. Therefore, an extension of the ODD is required to cater to such assistance in Level 3 and higher AVs.

### 2.1. ODD

The ODD was defined by the National Highway Traffic Safety Administration in 2017 [7]. This is the key to ensuring safety in the functional boundaries of an ADS, that is, an autonomous vehicle (AV) [8,9]. It is the domain under which an ADS, can operate properly [10]. The ODD varies according to AV levels. It includes the information required to define the capabilities or boundaries of ADSs, including roadway types, geographical area, speed range, environmental conditions for the safe operation of an ADS, and other domain constraints. Such information is required to ensure safer real-world operation of AVs. The object and event detection and response (OEDR) under the ODD defines the detection capabilities and immediate driving tasks of the AV under the relevant circumstances. Therefore, the OEDR functions are expected to be able to detect and respond to other vehicles, humans (e.g., pedestrians and cyclists), animals, and objects that may affect the safe operation of AVs [7,11]. The dynamic driving task of an AV should be able to perform the real-time functions required for safe operation under its ODD [11]. Traffic-gesture recognition is an important aspect of safe driving. The addition of the traffic-gesture recognition capability leads to the expansion of the ODD in AVs Level 3 and beyond. The present manuscript expands the ODD by introducing traffic-gesture recognition capability in Level 3 and higher AVs. 

### 2.2. Three-Dimensional (3D) Hand-Gesture Modeling

The significance of multifaceted applications of hand gestures has increased in the industry and research fields [12,13,14,15,16,17,18,19,20,21,22,23,24,25,26,27,28,29,30]. Computer vision, pattern recognition, and human-computer interaction (HCI) are among the popular areas that involve 3D hand-gesture modeling [31,32]. Various works have focused on hand models using different methods [32,33,34,35,36,37]. To address the issues associated with hands that interact with objects, a public challenge was organized by Armagan et al. (called HANDS’19) [38]. They provided the parameters of the MANO hand model [39] to the participants. It contained a wide variety of high-resolution 3D scans collected from 31 different subjects. The MANO models were realistic and low-dimensional, containing a non-rigid shape that changed with pose. The model was attached to the standard parameterized 3D body shape model: the skinned multi-person linear model (SMPL) [40]. SMPL-X is an extension of SMPL, which has computed a 3D model of the human body and hand poses, along with facial expressions to facilitate the analysis of human actions, interactions, and emotions [41]. Pavlakos et al. used a single monocular image to capture the major joints of the body, containing the full 3D surface of the face, hands, and body, and provided free access to the SMPL-X for research purposes. Osman et al. introduced the sparse-trained articulated human-body regressor (STAR) to overcome the limitations of the SMPL [42]. The STAR model had fewer model parameters than the SMPL model. Rong et al., along with the Facebook research team, presented FrankMocap [43], a motion capture system for estimating 3D hand and body motions.

### 2.3. Recent Developments in Traffic Gesture Recognition

Traffic police officers are trained to render specific hand gestures using their body poses to control traffic on the road. Therefore, researchers have used various artificial intelligence techniques to recognize these gestures. Broadly, two approaches can be employed for recognizing gestures: an on-body sensor-based approach, and a vision sensor-based gesture-recognition approach. In the on-body sensor-based approach, gestures are recognized using microelectromechanical system (MEMS)-based sensors. 

Accelerometers and gyroscopes are commonly used with on-body MEMS sensors for the estimation of poses and movements. Various vision sensor-based methods have been explored for real-time face detection, human tracking, and hand-gesture recognition. Some of them utilize the concept of recognizing spatial-temporal gestures. This represents the movement and orientation of the arm and palm. In 2004, Kang et al. demonstrated gesture recognition for video games [44]; they combined gesture spotting and recognition by considering the upper body parts focusing on the head, left hand, and right hand to estimate the pose of users. They first estimated human poses and recognized gestures in their recognition system. Pose estimation was performed via feature extraction using morphological operations. These extracted features were fed to the spatial classification module to estimate the pose using k-means clustering. 

Furthermore, the gesture recognition module accomplishes spotting and recognition tasks on behalf of the symbol sequences generated from the spatial classification module. This approach is based on vision sensors and provides an interface for the video game player. However, luminance issues (caused by vision sensors, season, weather, etc.), as well as less efficient ML, AI-based intelligent algorithms, computational complexity, latency, and hardware issues, have become practical limitations in the past decade. Therefore, an on-body sensor-based approach was chosen for gesture recognition. In 2010, Yuan et al. utilized the on-body sensor method for gesture extraction of the Chinese traffic police [45]. However, technological advancements in hardware, ML, and AI-based intelligent algorithms have improved the performance of vision-based approaches and made them superior to the approaches based around on-body sensors. In particular, for traffic gesture recognition in AV systems, vision-based approaches are more advantageous than on-body sensor-based approaches.

Guo et al. and Cai et al. presented Chinese traffic police gestures recognized in complex scenes [46,47]. They utilized the upper body based on a five-part body model, by considering the torso of the traffic police only (i.e., excluding the head, neck, and limbs from the upper body). Therefore, their method failed in a few cases, such as in the case of side view scenes of traffic police, when there was more than one traffic policeman in the scene, and when there was an unclear or blurry scene. In addition, the algorithm highly depended on the five-part body model; therefore, the performance was solely dependent on the viewing angle. This meant that for the same scene, if the viewing angle changed, the prediction may have become incorrect.

Le et al. utilized depth images for the recognition of traffic-control gestures [48]. They captured the depth images of traffic police control gestures and constructed a human skeleton using a kinematic model. They utilized the joint angles of the human skeleton as the feature vectors to recognize different traffic gestures, using a support vector machine (SVM)-based classifier. Sathya et al. performed different experiments using decision trees (DTs), random forests (RF), and SVM to recognize traffic gestures [49]. They compared these three classification approaches on a real-time traffic gesture dataset, and reported that RF had a higher classification performance than SVM and DT. In [50], Guo et al. utilized the fusion of the static and dynamic descriptors of Chinese traffic police to recognize their hand traffic gestures. Ma et al. suggested a spatiotemporal convolution neural network for real-time traffic gesture recognition [51]. 

In [52], an HCI-based gesture-learning approach was presented to understand humans on the road. The GLADAS gesture-learning method was designed in a simulated environment to teach AVs to understand pedestrian hand gestures. Chen et al. utilized a semi-supervised learning-based SO-HandNet model to estimate the 3D hand poses [53]. SO-HandNet is an autoencoder-based self-organizing network. They used a three-step process in a pipeline to estimate the 3D hand poses. The hand feature encoder in the first step was used to extract multi-level features from the hand point cloud, and a hand-pose estimator in the second step was used to fuse them to the 3D hand poses. In the third step, a hand feature decoder was used to recover the input point cloud from the encoded feature. Al-Hammadi et al. introduced a 3DCNN approach for hand-gesture recognition in sign language applications [54]. The 3DCNN model was trained for the region-based spatiotemporal features of hand gestures. He et al. [1] used handcrafted features along with a convolutional pose machine (CPM) to recognize eight types of Chinese traffic police gestures in real time. They modified the CPM for the extraction of spatial features and the long short-term memory network for the extraction of temporal features. Wiederer et al. introduced a dataset for traffic-control gesture classification [2]. It consisted of a 3D body skeleton input of five individuals of different body types. It had 250 sequences, ranging from 16 s to 90 s per sequence.

Most of the approaches involved in traffic gesture recognition utilize hand-gesture recognition techniques. However, the inefficiency of such approaches in the context of AVs has two main causes. One is that the hand gesture algorithm applied is trained for any human, and is not specific to the traffic controller. The other is that the traffic police, in general, wear gloves on their hands during traffic control. Therefore, efficient traffic gesture recognition systems should be able to recognize an ATC and their hand gestures with gloves.

### 2.4. Commonly Used Traffic Control Hand Gestures

Different countries use different styles of hand gestures to control traffic. Furthermore, ATCs wear a different dress according to the rule of a particular country. 

**Figure 4:**
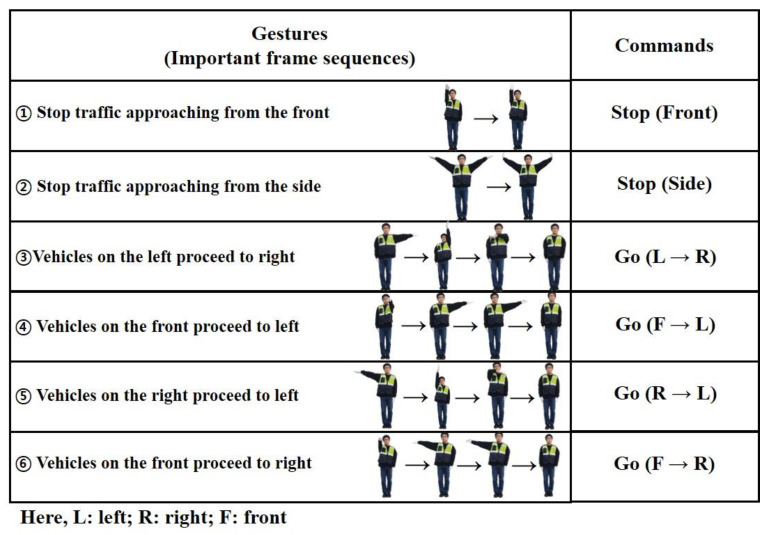
Traffic control hand gestures. These are a combination of commonly used traffic control gestures in different countries.

However, the gestures vary slightly. In addition, almost everywhere, ATCs wear gloves on their hands. We compared the traffic control hand gestures used by the ATCs of a few countries. Such as India (HAND SIGNALS. Available online: https://www.ctp.gov.in/HandSignals.htm#Driver_Hand_Signals (accessed on 2 August 2021), United Kingdom (The Highway Code: signals by authorized persons. Available online: https://assets.publishing.service.gov.uk/media/560aa62bed915d035c00001b/the-highway-code-signals-by-authorised-persons.pdf (accessed on 2 August 2021)), China [1], and Singapore (Understanding A Traffic Cop’s Hand Signals. Available online: https://aa-highway.com.sg/understanding-a-traffic-cops-hand-signals (accessed on 2 August 2021).)). By observing these symmetrical traffic control hand gestures followed in various countries, we generalized commonly used traffic control hand gestures of ATCs worldwide, as shown in Figure 4. The commonly used traffic control hand gestures with their meaning and the abbreviations used by us are described in Figure 4. Here, the meaning of the gestures is considered from the viewpoint of an ATC.

## 3. Methods

We proposed a traffic gesture recognition technique by applying 3D modeling of the hand pose. For efficient traffic gesture recognition, the approach must eliminate non-authorized hand gestures from authorized traffic-control hand gestures. We developed an ATC hand-gesture recognition system. We focused only on the gestures of ATCs. This eliminated any chance of misinterpretation caused by other hand gestures to the AV. The proposed method consisted of three steps:

1. ATC detection;
2. ATC’s hand and palm detection and 3D hand modeling;
3. Traffic control hand gesture recognition.

Section 3.1 highlights the stages of our proposed ATC hand-gesture recognition system, along with a description of the 3D hand modeling. Section 3.2 and Section 3.3 explain the classifier involved and its training and inference mechanism, respectively.

### 3.1. ATC Hand Gesture Recognition System

**Figure 5:**
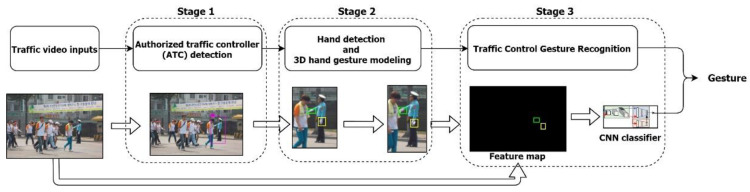
Block diagram of the ATC hand gesture recognition system.

Our proposed traffic control hand gesture recognition system involved ATC detection and hand-gesture recognition. It had three stages for accurately estimating traffic control hand gestures. The first stage detected the potential traffic controller from the visual data. An object detection model separated the potential person from the others present in the scene. In the second stage, the detected hand gestures of the authorized persons were modeled in 3D. This combined gesture modeling described the traffic-control required for AVs; it behaved as an extension of the ODD. A block diagram of the proposed approach is shown in Figure 5. The first problem of recognizing an ATC is resolved in the first stage. The next problem of correct recognition of traffic control hand gestures is resolved using the other two stages. Stage 2 involves 3D hand modeling, and stage 3 involves gesture recognition.

**Figure 6:**
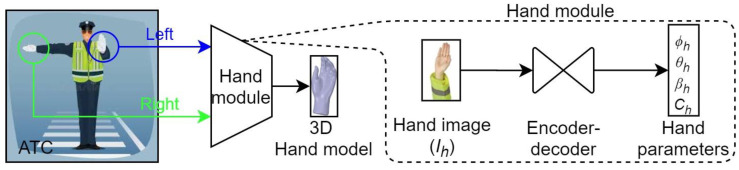
Scheme for 3D hand modeling of the traffic control hand gestures.

Hand modeling was performed using the following scheme. For a given image of the ATC, hand images (for left and right hands) were detected for 3D hand modeling. The hand module was used to model hand gestures into 3D hand models. The hand-gesture modeling scheme is shown in Figure 6.

We used the FrankMocap model for 3D hand modeling. It uses monocular inputs and the parametric model of SMPL-X for 3D hand modeling of faster monocular hand and body motion. The hand of SMPL-X is generated by a differentiable function (for body, face, and hands) $M\;\left(\theta ,\;\beta ,\;\psi \right):\;{\mathbb{R}}^{\left|\theta \right|\times \left|\beta \right|\times \left|\psi \right|}\to {\mathbb{R}}^{3N}$. Here, *N* is the number of vertices, *β* is the shape parameter, and *θ* is the pose parameter for the body, face, and hands. *ψ* is a facial expression parameter. The complete function is defined as follows:

(1) $$M\;\left(\theta ,\;\beta ,\;\psi \right)=W(T_{P}\left(\left(\theta ,\;\beta ,\;\psi \right),\;J\left(\beta \right),\theta ,\;\omega \right)$$

(2) $$T_{p}\left(\theta ,\;\beta ,\;\psi \right)=\bar{T}+B_{S}\left(\beta ;S\right)+B_{E}\left(\psi ;\;\varepsilon \right)+B_{P}\left(\theta ;P\right)$$

Here, $W\left(T_{P},\;J,\theta ,\;\omega \right)$ denotes a standard linear blend skinning function, J(β) is a function of body shape, and ω is the blend weight. $B_{S}$ is the shape blend shape function, $B_{E}$ is the expression blend shape function, $B_{P}$ is the pose blend shape function, and $\bar{T}$ is the template mesh defined in SMPL. 

We used a similar approach to define the hand module ($M_{H}$). In Figure 6, the hand image ($I_{h}$) is fed to the hand module ($M_{H}$) to obtain a 3D hand model. Our hand module is defined by Equation (3) as follows:

(3) $$M_{H}\left(I_{h}\right)=\left[\emptyset_{h},\theta_{h},\beta_{h},C_{h}\right]$$

Here, $\emptyset_{h}$ is the global orientation of hand ($\emptyset_{h}\in {\mathbb{R}}^{3}$); $\theta_{h}$ is the hand-pose parameter ($\theta_{h}\in {\mathbb{R}}^{3\times 15}$); $\beta_{h}$ is the shape parameter ($\beta_{h}\in {\mathbb{R}}^{10}$); and $C_{h}=\left(t_{h},s_{h}\right)$ is the hand region defined by the scaling factor $\left(s_{h}\in \mathbb{R}\right)$, and the 2D and translation of the hand image plane $\left(t_{h}\in {\mathbb{R}}^{2}\right)$. The hand module has hand mesh vertices ($V_{h}\in {\mathbb{R}}^{778\times 3}$). The 3D joint regression function for the hand is $\left(J_{h}^{3D}\in {\mathbb{R}}^{21\times 3}\right)$. It is defined by the regression matrix of hand $R_{h}$. Equation (4) represents the definition of $J_{h}^{3D}$, as follows:

(4) $$J_{h}^{3D}=R_{h}\left(V_{h}\right)$$

The translation of the *i*th 3D hand joint to a 2D hand joint is achieved by the orthographic projection (П) of the 2D translation $t_{h}$ using a scaling factor $s_{h}$. It is defined as follows:

(5) $$J_{h,i}^{2D}=s_{h}\prod \left(J_{h,i}^{3D}\right)+t_{h}$$

The overall loss function (${ℒ}_{Overall}$) of hand model training is defined by Equation (6). It is a combination of different losses, such as hand-pose loss (${ℒ}_{\theta }$), 3D key-point loss (${ℒ}_{3D}$), 2D key-point loss (${ℒ}_{2D}$), and regularization loss (${ℒ}_{R}$). This combination requires the corresponding weights (ω) to adjust the error. It is given as follows:

(6) $${ℒ}_{Overall}=\omega_{\theta }{ℒ}_{\theta }+\omega_{2D}{ℒ}_{2D}+\omega_{3D}{ℒ}_{3D}+\omega_{R}{ℒ}_{R}$$

(7) $${ℒ}_{\theta }=\|\emptyset_{h}-{\hat{\emptyset }}_{h}{\|}_{2}^{2}$$

(8) $${ℒ}_{2D}=\|J_{h}^{2D}-{\hat{J}}_{h}^{2D}\|$$

(9) $${ℒ}_{3D}=\|J_{h}^{3D}-{\hat{J}}_{h}^{3D}{\|}_{2}^{2}$$

(10) $${ℒ}_{R}=\|\beta_{h}{\|}_{2}^{2}$$

The losses were obtained using the squared error function. Here, ${\hat{\emptyset }}_{h}$, ${\hat{J}}_{h}^{2D}$, and ${\hat{J}}_{h}^{3D}$ are the ground truth annotations of the hand pose, 2D key-points, and 3D key-points, respectively. The 2D key-points provide the camera projection parameters. The Algorithm 1 is as follows:

**Definitions** **1.** *Authorized traffic controller (A); hand (H); 3D hand model (M); previous frame (f1); pose frame (f2); feature extraction (E); E_f1_ & E_f2_ are the extracted features corresponding to f1 & f2; contrastive embedding (* C *); single inference (* I *); gesture (* G *).*

**Algorithm 1: ATC Hand Gesture Recognition**
**Functions:** ℱ = object detector; ℋ = hand detector; ℳ = 3D hand model; E = feature extractor; C = contrastive embedding; 𝔗 = single inference.Input: *videoframes* (*onboard device*)  *A* = ℱ(*Input*)  // ATC detection  *for* each hand in *f1* & *f2*:  *H* = ℋ(*A*)  // Hand detection  *M =* ℳ(*H*)  // 3D hand modeling  *E* = E(*M*)  // Hand feature extraction  *E* = {*E_f1_*, *E_f2_*}  // Stores hand features (of *E_f1_*, and *E_f2_*)  C = C(*E*)  // Contrastive embedding calculation for *E_f1_*, and *E_f2_*  I = 𝔗(*E_f2_*)  // Inference for
*E_f2_*  $G=\{\begin{array}{c} C+I,\;if\;poses\;have\;difference\\ I,\;if\;poses\;have\;no\;difference \end{array}$return (G)

### 3.2. CNN Classifier for Gesture Recognition

**Figure 7:**
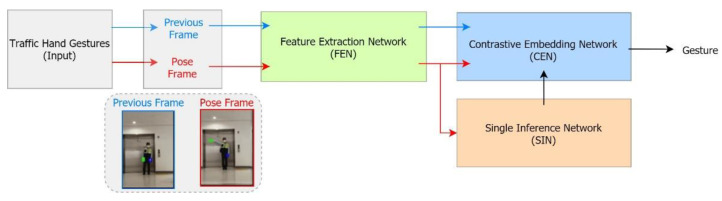
Proposed CNN classifier architecture. It uses FEN to extract features and, by combining results of CEN and SIN, recognizes the traffic-control gestures.

Our proposed ATC hand-gesture recognition system estimated traffic control hand gestures using our CNN classifier. It consisted of four networks: the feature extraction network (FEN), the feature decode network (FDN), the contrastive embedding network (CEN), and a single inference network (SIN), as shown in Figure 7.

We used two frames (i.e., the previous frame and the pose frame) sampled per second in our hand-gesture recognition classifier. Here, the previous frame represented the frame of the ATC just before the traffic control hand gesture, and the pose frame was the frame in which any traffic control hand gesture was performed by the ATC. The FEN extracted the corresponding features present in both frames. It had three hidden layers with 16, 32, and 64 filters. It was a pre-processing network, that received the input images (of size 128×128×3) and extracted the features (of size 32×32×64). Furthermore, it fed these features on the CEN. The extracted features were fed to the feature decode and contrastive embedding networks. The FDN was the transpose of the FEN, and was used for the reconstruction of the frames. It was a decoder network that received the produced feature map from FEN as an input (of size 32×32×64) and restored it to the original image (of size 128×128×3). The CEN had three fully connected (FC) layers (64, 100, and 6 filters). The features extracted by the FEN for both images (previous frame and pose frame) were fed to the CEN. By subtracting the 64-dimensional feature maps of these two inputs, the difference (contrastive loss) was utilized to classify the gestures. It estimated the contrastive loss between the previous frame and pose frame, in terms of the extracted features obtained from the FEN (i.e., 64-dimensional feature vectors for each frame).

For a pair of images $\left(I_{1},\;I_{2}\right)$, the contrastive loss function ($L_{Contrastive}$) is defined by Equation (11), as follows:

(11) $$L_{Contrastive}\left(I_{1},\;I_{2}\right)=\left(1-S\right)\frac{1}{2}{\left(ED\right)}^{2}+\left(S\right)\frac{1}{2}{\left\{max\left(0,\left(m-ED\right)\right)\right\}}^{2}$$

Here, m is a positive value that provides a margin around the embedding space. Only those dissimilar feature vectors of the image pairs can contribute to the loss function whose distance is within this margin value. The ED is the Euclidean distance (parameterized distance) between the feature vectors of image pairs $\left(I_{1},\;I_{2}\right)$. The similarity measure S is given by Equation (12). Where:

(12) $$S=\{\begin{array}{c} 1\;if\;I_{1}≅I_{2}\\ 0\;otherwise \end{array}$$

A SIN (consisting of three FC layers of sizes 32, 50, and 6) and a CEN were used for the prediction of the current hand gesture. The SIN network was additionally used for inference with only the current frame. It had the same structure as that of the CEN, except that a single frame was included, and the number of parameters was approximately half. The output was the traffic control hand gestures, as shown in Figure 4.

### 3.3. Training and Inference

The training and inference of the proposed ATC hand gesture recognition classifier CNN are shown in Figure 7. The training of the classifier CNN has four blocks: FEN, FDN, CEN, and SIN. The FEN was a pre-processing auto-encoder network. It required two frames (i.e., the previous frame and the pose frame) sampled per second. It extracted 64-dimensional feature vectors (or feature maps) from both frames. The FDN worked as a decoder network that received the previously produced feature map as an input, and restored it to the original image frame. This enhanced the performance of the FEN.

The feature decoder network and the reconstructed frames eventually served as an additionally attached network to learn the FEN required for inference, and as a label for learning the FEN using this structure. The mean squared error was used as the loss function for training the FEN and the FDN, with a learning rate ($l_{r}$) of 0.001. However, the cross-entropy loss function was utilized to train the CEN and SIN with a learning rate ($l_{r}$) of 0.0001. In the CEN, the input was the 64-dimensional difference between the features of the previous frame and the pose frame captured with a frame gap of one second. It was used to predict the traffic control hand gestures shown in Figure 4. The training process was performed in two steps. First, the FEN and FDN were trained to obtain 64-dimensional feature vectors of both frames. Then, the obtained parameters of the FEN were kept frozen, while the training of the CEN continued. This was to maintain an intact prediction.

Only three networks were used for the inference. FDN was not required in the inference process. We used SIN along with the CEN for traffic gesture prediction. The CEN required two frames sampled per second to accurately predict the hand traffic gestures. It utilized the difference between the two frames for gesture prediction. Common traffic control hand gestures were performed within one second; this was the reason for the number of sampling frames per second. However, few gestures were very quick, and some did not involve any change within one second. In such cases, both frames (i.e., the previous and pose frames) were almost the same. Therefore, there was no difference perceived by the CEN, and eventually predicted no gestures. A SIN was then added to avoid such mistakes. It worked when there was very little or no difference between the previous frame and the pose frame. This required a single frame (pose frame) for inference.

## 4. Results and Discussion

**Figure 8:**
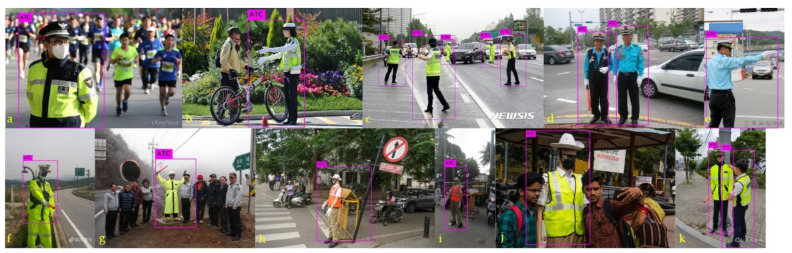
Different scenes (**a**–**e**) show detections of ATCs through the proposed approach. Special cases are shown from (**f**–**k**).

Section 2.4 states that there is only a slight difference in traffic control hand gestures in different countries. We sorted the symmetrical traffic control hand gestures in Figure 4. Our proposed method classified these gestures accurately in agreement with their real inference by a human. In stage one of our proposed method, we first determined the ATC from the scene and then applied 3D hand modeling to recognize the gestures in stage two. ATC detection using the proposed algorithm is shown in Figure 8. We used a variety of scenes publicly obtained from different websites to validate our proposed ATC detection approach.

Figure 8a,b show ATCs with other humans in the same scene. Figure 8c shows multiple ATCs in the same scene. Our algorithm correctly detected the ATCs in each of them. Figure 8d shows the best drivers; best drivers are recognized as authorized volunteer traffic controllers in South Korea. Therefore, they were also detected as ATC. Similarly, Figure 8f–k represent special situations in which humans, ATC, and mannequin ATC are in the same scenes. Our proposed ATC detection approach could recognize ATCs in such scenarios, as well. The mannequin in Figure 8f also represents an ATC to control traffic on the highway. Therefore, the ODD should be recognized correctly. In our approach, it was also detected as an ATC.

**Figure 9:**
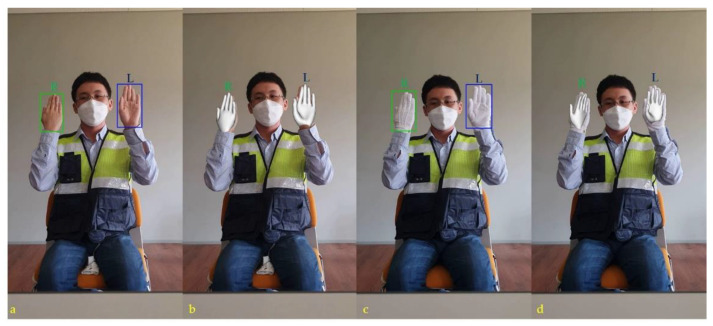
Left (**L**) and right (**R**) hand–palm detection. (**a**) Left and right hands without gloves; (**b**) the corresponding 3D hand model. (**c**) Left and right hands with gloves; (**d**) the corresponding 3D hand model.

**Figure 10:**
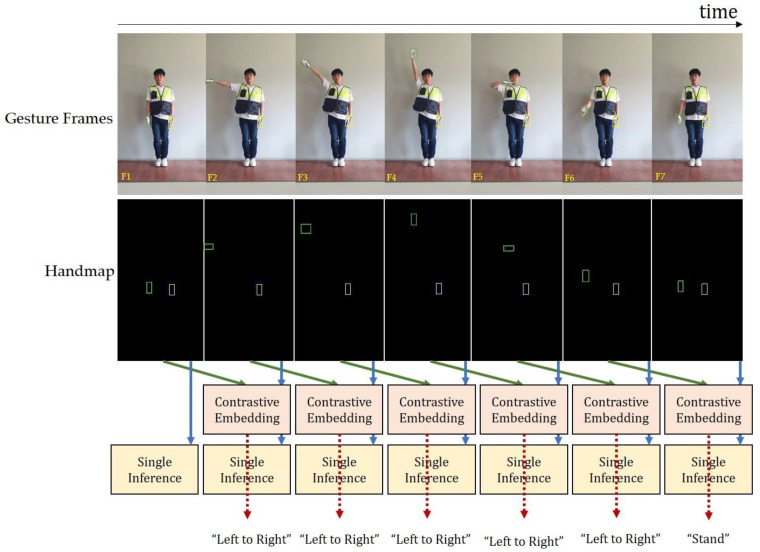
Frame-by-frame hand gesture recognition in stage 3 of the proposed approach. In this particular example, seven frames (**F1**–**F7**) have constituted a complete “left to right” traffic control hand gesture. Each gesture recognition is achieved by combining the contrastive embedding and single inference predictions.

In stage two, hand gestures were modeled in 3D. The 3D modeling of the hands with and without gloves is shown in Figure 9. It discerns the correctly modeled left and right hands in 3D. The gloves impose difficulty in recognizing the front and back sides of the palm of the hand. The side of the hand plays a significant role in traffic control hand gestures; therefore, 3D modeling of the hand provides meaningful hand gestures that are easier to recognize. In stage three, traffic control hand gestures were recognized using our proposed approach. Figure 9 shows the procedure involved in stage three of the hand-gesture recognition approach. A SIN uses a single frame to predict traffic gestures. However, the CEN requires two frames sampled per second for gesture prediction. The same is illustrated in Figure 10, by considering the “left to right” traffic control hand gesture. Different instances show the prediction steps through SIN and CEN. Each traffic gesture had multiple frames. Therefore, SIN alone cannot predict the correct recognition. However, the CEN required two frames in its prediction, which were unavailable at the start of the gesture. Therefore, in the case of the F1 frame, the prediction was discerned only by SIN and not by CEN. CEN started its prediction after the starting frame of the gesture. The FrankMocap provided a 3D hand model, used for the estimation of the hand-map.

A hand-map was obtained using the object detection method. As depicted in Figure 10, contrastive embedding is predicted using two hand-maps (the current frame and the previous frame captured by approximately 0.625 s/frame) as inputs. The single inference provided predictions using the hand-maps of the current frame as inputs. A complete gesture was recognized by combining the results; therefore, in this example, the predicted gesture was left to the right. We considered multiple input frames to validate the efficacy of our approach for traffic gesture recognition. The training dataset consisted of a video of 40 min and 34 s, whereas the test dataset had 24 min and 30 s of video length. All videos were captured at a 24 frames per second (24 fps) frame rate. According to the aforementioned traffic gestures (Figure 4), we considered 9966 frames for stop gestures from the front and 3740 frames for stop gestures from the side. Similarly, the number of frames for four different possible turns (i.e., L → R; R → L; F → L; and F → R) were 4841; 4121; 6528; and 6095, respectively. Thus, we had a total of 35,291 input frames containing different traffic gestures.

Figure 10 shows the recognition results of the traffic gestures using individual and combined frames. As depicted, the recognition error mostly occurred during the start or end of the gestures. Therefore, there were errors in the recognition frames; however, the complete gesture was recognized accurately. It should be noted that every traffic control hand gesture required a different number of frames, mainly because each gesture has different steps in its completion (from the start of the gesture to the end of the gesture, as illustrated in Figure 10). This difference varies for the same person, for the same gesture. Therefore, the frame-wise recognition of traffic gestures is inappropriate. Instead, complete gesture recognition is a better way to recognize traffic gestures.

Table 1 presents the efficiency of the gesture recognition for each frame. Our approach showed 96.70% accuracy in correctly recognizing frames consisting of traffic control hand gestures. Here, the given accuracy is per frame, although all (complete) traffic gestures were recognized correctly.

**Figure 11:**
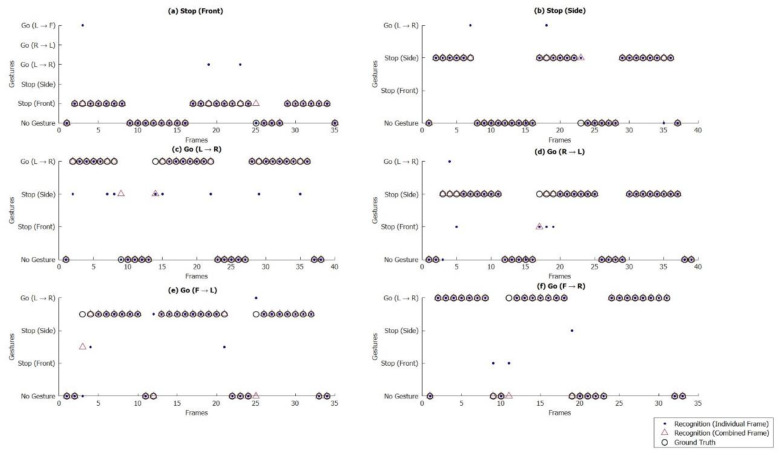
Frame-by-frame gesture recognition for the considered hand gestures. Individual frame recognition represents the detection using SIN, and the combined frame recognition shows the combined result of CEN and SIN for each frame.

**Figure 12:**
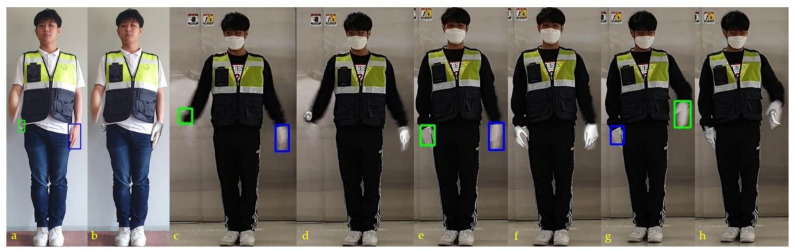
Several examples illustrating errors in the recognition of frames. Here, (**a**,**c**,**e**,**g**) represent hand detection, (**b**,**d**,**f**,**h**) represent the corresponding 3D hand modeling.

Error in the recognition of the frame is shown in Figure 11. The shown frame had one missing hand. This frame captured the moving right-hand traffic gesture. Because of the start of the gesture and the motion, that frame missed the correct image of the right hand. Therefore, the proposed SIN and/or CEN networks have errors in the prediction of gestures in such a frame. Eventually, the combined results reflected an error. However, the proposed method correctly recognized every complete traffic gesture (as depicted in Figure 12).

**Figure 13:**
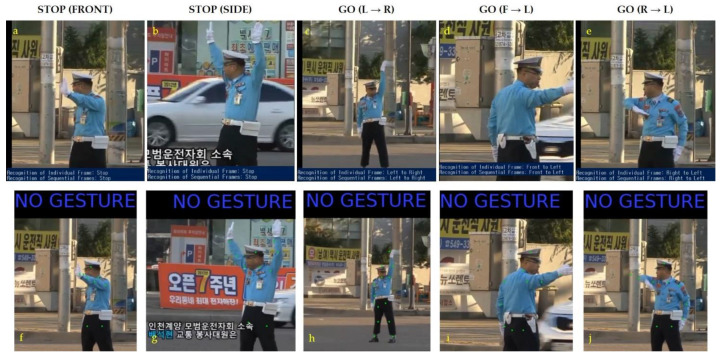
Performance comparison of the traffic gesture recognition for different traffic gestures. Here, (**a**–**e**) represent gesture detection by our proposed approach, and (**f**–**j**) represent the corresponding detections through the CPM approach.

A comparison of traffic gesture recognition is depicted in Figure 13. We considered a public domain dataset (https://youtu.be/Z987tL5XZbE (accessed on 9 November 2021)) to compare the performance of our proposed approach with that of the CPM approach given in [1]. Furthermore, the reported accuracy of the “STOP (Front)” gesture of [1] was 94.81%, whereas ours was 99.27%. Similarly, the reported accuracy of the “GO” gesture of [2] was 87.3%, whereas ours was 95.36% (it is the average accuracy of the GO gestures).

As shown in Figure 13, by judging the traffic controller’s commands, the posture of the human body results in the inaccurate recognition of gestures. This is because hand–palm movement is the most important parameter in traffic controller commands. We applied the same method (recognizing hand–palm movement) in our proposed approach. Figure 5, Figure 6 and Figure 7 explain the proposed approach.

Our proposed approach had three deep-models in three stages. We used an embedded board (Rockchip RK3399Pro board) for our autonomous driving (AD) experiments. For an AD system, a lightweight and efficient model was of the utmost importance. The computational cost of our entire system, in terms of the time required to perform individual operations in our proposed approach, is as follows:

1. ATC detection time (in seconds): 0.138 s;
2. Hand–palm detection and 3D modeling time (in seconds): 0.250 s;
3. Traffic command recognition time (in seconds): 0.044 s.

A total of 0.432 s was required to perform each task using the onboard device (here, RK3399Pro). Furthermore, we used two frames (the previous frame and the pose frame, with a time lapse of 0.625 s between these two frames) to produce the results. Therefore, the total time required for the normal operation of the proposed system was approximately 0.625 s. Therefore, it was possible to perform all operations normally in this environment.

## 5. Conclusions

Vision-based traffic control hand-gesture recognition suffers from multiple issues. Two of the main reasons are the on-road presence of humans other than ATCs, and the use of gloves in traffic control hand gestures. The present work incorporates an important stage of ATC detection. This method has two main advantages. First, our proposed traffic gesture recognition system had no problem with persons imparting hand gestures on roads other than ATCs. Second, AVs have a human-level understanding of situation-aware traffic gesture recognition, even under irregular situations. Furthermore, the 3D hand modeling approach incorporated in our proposed system had the advantage of correct gesture recognition, even with a gloved hand. It had the additional advantage of correct recognition of the palm, which plays a very important role in the understanding of traffic gestures. Furthermore, we utilized a combination of CEN and SIN in our classifier for frame-by-frame gesture recognition, which ensured complete gesture recognition. Therefore, it can be considered as an extension of the ODD for AVs of Level 3 and beyond. A future extension could be achieved by incorporating hand-gesture recognition of pedestrians and cyclists.

## Figures and Tables

**Table 1:** Recognition efficiency of common traffic control hand gestures by our CNN classifier.

Gestures	Stop (Front)	Stop (Side)	L → R	R → L	F → L	F → R
Input Frames	9966	3740	4841	4121	6528	6095
Error	72	103	182	231	219	356
Recognition (%)	99.27	97.25	96.24	94.39	96.65	94.16

Here, L: left; R: right; F: front.

## Data Availability

The image data used to support the findings of this study are included in this article.
